# Behavior Identification Based on Geotagged Photo Data Set

**DOI:** 10.1155/2014/616030

**Published:** 2014-02-24

**Authors:** Guo-qi Liu, Yi-jia Zhang, Ying-mao Fu, Ying Liu

**Affiliations:** Software College, Northeastern University, Shenyang 110819, China

## Abstract

The popularity of mobile devices has produced a set of image data with geographic information, time information, and text description information, which is called geotagged photo data set. The division of this kind of data by its behavior and the location not only can identify the user's important location and daily behavior, but also helps users to sort the huge image data. This paper proposes a method to build an index based on multiple classification result, which can divide the data set multiple times and distribute labels to the data to build index according to the estimated probability of classification results in order to accomplish the identification of users' important location and daily behaviors. This paper collects 1400 discrete sets of data as experimental data to verify the method proposed in this paper. The result of the experiment shows that the index and actual tagging results have a high inosculation.

## 1. Introduction

With the popularization and development of mobile devices, the speed of producing photos by people has become faster and faster. Now people often use Flickr, Instagram, or other photo sharing softwares to manage and store these photos. Because these photos are fragmented, users do not sort or classify them so that people always waste time searching the photo storage when browsing. Therefore, how to automatically classify enormous photos is an urgent problem to be solved. And at the same time people want to figure out user's behavior rules according to the relationship between the user's behavior and important location in the classification results. This is one of the heated issues of social computing researches at present.

Now many scholars take part in social computing researching on identification of user's behavior rules and important location. At the initial stage, most of the researchers use the automatic positioning and sensing equipment to collect the user's mobile information [1, 2] or infer the user's mobile route from the signal of wireless network [3, 4]. With the automatic and continuous way of data collection, identification of important location and behavior needs a long time to analyze the movement of the user with high cost and ignores the protection of user's privacy which leads to some restrictions to the follow-up research. Relatively, the gaining of the identification through discrete geotagged photo is more convenient with great quantity. This paper focuses on the photos offered by some photo sharing software similar to Instagram and Flickr. The photos have some data information such as time stamp, location information, and text label, which are called geotagged photo [5] data set.

Geotagged photo data in this paper are collected individually for 16 months (http://202.118.31.219/FindMe) containing photos, shooting position information, and time stamp. According to aforementioned characteristics, this paper proposes a multiple classification method. First, dividing the data set into several sets based on the different shooting cities; second, further dividing the data set based on crucial shooting spots; finally, classifying the result according to users' behavior, thus a three-layer structure like “city-place-behavior” is figured out. Considering the overlap of the behaviors' meaning and the complexity of the photos' information, some tags as an index are given to the data with high possibility from kinds of classification results; in this way, the goal of sorting the data set is achieved.

This paper is organized as follows.  Section 2 describes related work.  Section 3 explains the multiple classification methods and related definitions.  Section 4 presents each phase of the experiment process and result.  Section 5 is the conclusion.

## 2. Related Work

Now, in the social computing research area, there are some researchers that choose the continuity mobile data to get human's behavior and movement mode. Reference [6] uses DBSCAN algorithm to extract the areas where users upload photos frequently, which are defined as appealing areas in which some significant research will be conducted. Reference [7] uses support vector machine (SVM) to train data to get the photos which can represent the areas. Reference [8] uses an algorithm based on density to divide the area into grids to get the dense connected region. Reference [9] proposes a multidisciplinary method for the recognition of physical activity with the emphasis on feature extraction and selection processes, which are considered to be the most critical stages in identifying the main unknown activity discriminate elements. References [10, 11] identify users' important places and behaviors through their long-term continuous GPS data (Rekimoto et al.). Reference [4] used the continuous location information uploaded by wireless network to get users' location and movement mode. However, the high cost, the limited development of application, and disrupting users' privacy are the fatal disadvantages of the method. Reference [12] tries to analyze the photos from mobile and compared to the known scenic then predict the user location and accomplish the route tracking according to the time and spatial information. And the geotagged photo data set used in this paper is uploaded by users themselves with a diversity of information, which is more conducive to research.

The collected data need to be divided into multiple classifications based on users' places and behaviors. Now, the SVM classification algorithm [13] and RBF neural network [14] are usually used. We use LIBSVM [15] and the neural network tool supplied by Matlab to classify the data.

## 3. Solving Method and Related Definitions

The data this paper collected is uploaded by the users, so filtering the noise for the data set is not necessary. Classification algorithms are used to divide the data set, and the class labels consisted of users' important places and behaviors, so we can identify user's important places and behaviors by the classification result.

### 3.1. Classification Process

**Figure 1:**
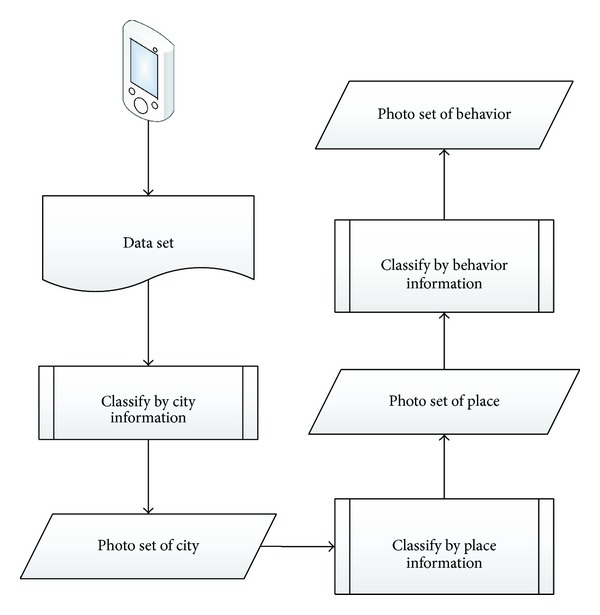
Classification flow chart.

Figure 1 shows the classification process in this paper, it has 3 steps: “city” classification, “important places” classification, and “behavior” classification.

*“City” Classification*. We can infer user's behavior by the city where he is. The behaviors may be different for the difference between the city he lives and the city where he is traveling to or on business in. So we can divide the data set into 2 parts: one is the living part, and the other is the traveling part, then we can divide the living part in detail.

*“Important Place” Classification*. This step divides the living part by user's important shooting places, like office, home, and restaurant. This work differentiates the same behaviors happening in different places.

*“Behavior” Classification*. This step divides the data set by user's behaviors. The data set only contains time stamp and location information that can be used by classification algorithm, so we cannot divide the data set into many classes. There are only a few branches like “work,” “eating,” “go out,” and so on.

**Figure 2:**
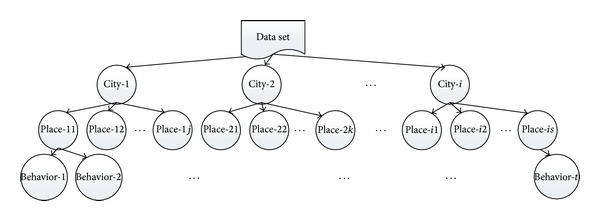
Classification chart.

Through the above 3 steps, the data set is divided into several classes which consisted of “city,” “important place,” and “behavior”. The overall classification result is shown in Figure 2.

### 3.2. Label Allocation

In general classification method, the classification result of each data is unique, but the unique result will lose some important information in the classification of geotagged photo data set. For instance, if the user goes out to do some work and then has a meal with clients, the data recording this event should contain both going out for a meal and going out for work; however, no matter which class this data is to be divided into, the other behavior information will be lost. So in order to keep the information intact, this paper adopts a method of the probability estimation. If the probability is higher in one classification than the threshold, we assign this class a label. As a result, each datum may have several labels.

When all data is labeled, an index is built. The classification of the data set and getting locations' information and behaviors' information together are achieved. It is more accurate to assume the users' behaviors when they are uploading new data.

### 3.3. The Definitions

This section formally defines the concepts in this paper.

**Definition 1 (city)**

It means the city where a user takes the photo. This information can be got from the GPS information of our application.

**Definition 2 (place)**

It means the location where a user takes a photo, such as school, home. It also can be automatically accessed from our application.

**Definition 3 (behavior)**

It means the behavior that the photo records.

**Definition 4 (label)**

It consists of the three definitions mentioned above:

(1) $$\begin{array}{c} \text{Labe}{\text{l}}_{\text{inm}}=\left\{\text{Cit}{\text{y}}_{i},\text{Plac}{\text{e}}_{n},\text{Behavio}{\text{r}}_{m}\right\}. \end{array}$$

Each label consists of “city,” “place,” and “behavior”; for example, the user has a meal in a restaurant in a city.

**Definition 5 (probability_label_)**

It means that the estimated probability of the data belongs to the class, if Probability_label_ ≥ *τ*, *τ* ∈ (0,1] one considers this label of the data effective, and it is assigned to the label set of data.

**Definition 6 (LabelSet_photo_)**

It consists of the labels which are assigned to the data:

(2) $$\begin{array}{c} \left(\text{Label},\text{Probability}\right)\in \text{LabelSe}{\text{t}}_{\text{photo}}. \end{array}$$

## 4. The Classification Process and Result

### 4.1. Data Acquisition and Explanation

**Figure 3:**
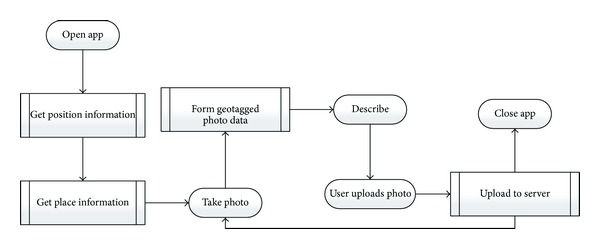
Collection process and data form.

The data in this paper is collected by the iPhone app developed by our lab. This app has photographed function and positioning function. When user takes a photo the app will automatically combine the photo, time information, and location information into a geotagged photo data. Users can upload the data into our lab's server. This data set has more than 1400 records, collecting for 16 months. The collection process and data form are shown in Figure 3.

### 4.2. Experimental Result

#### 4.2.1. Initial Experimental Result

**Figure 4:**
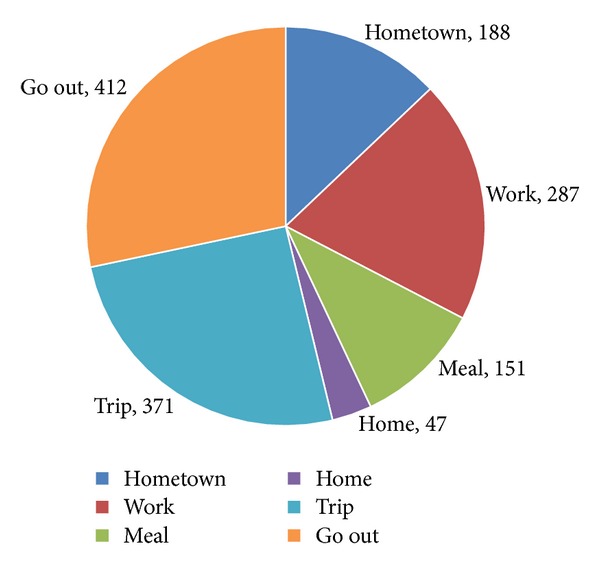
Number of each class.

At the beginning of the experiment, data are divided into 6 classes according to the analysis of data set. They are “work,” “meal,” “go out,” “home,” “trip,” and “hometown”. We give each record a label to define which class it belongs to. The number of each class is shown in Figure 4.

**Figure 5:**
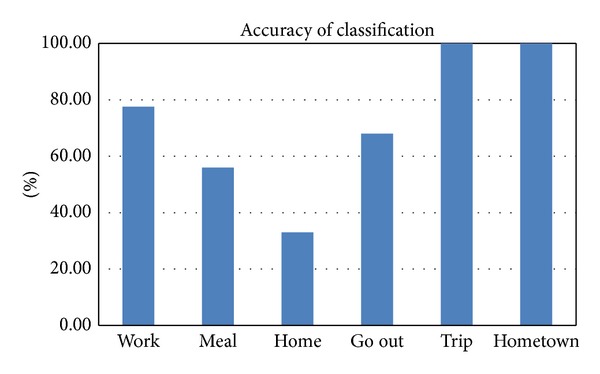
The accuracy of classification.

In the process of training, we use 5 cross validations to reduce errors and the accuracy is the average of the 5 results. The classification algorithms we used are SVM and RBF neural network. The accuracy of SVM is 77.59%, and the accuracy of RBF neural network is 71.72%. Based on the results, the accuracy of SVM is higher. So we choose to analyze the result of SVM classification result. As shown in Figure 5, the accuracy of “trip” and “hometown” is 100%. It is because we first divide the data set by the city information before the classification. The accuracy of “meal” and “home” is low, because the data of “home” is few and the data of “meal” is discrete in position, like “go out”.

We can know that the above method has some problems. The classes both have “home” which means place and “meal” and “work” which means behavior; the classification algorithm cannot work well, so we decide to use the multiple classifications method.

#### 4.2.2. Multiple Classification

**Figure 6:**
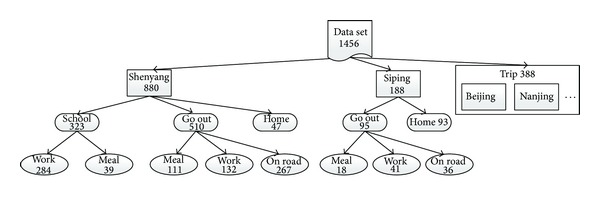
The structure of multiple classifications.

The structure of multiple classifications and the number of record of each class are shown in Figure 6.

**Figure 7:**
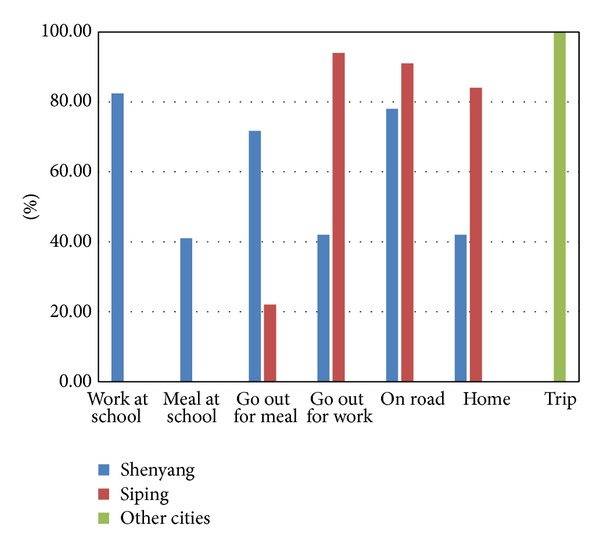
The accuracy of the multiple classifications.

The accuracy of the multiple classifications is shown in Figure 7.

By using the multiple classifications method, the accuracy of the data in Shenyang is 71.7%, and the accuracy of the data in Siping is 82.4%, not including the data of “trip” class. It is clearly shown that the whole accuracy has been improved. But there are still some problems. We find that just one class's label cannot show all the significance of a data, and it may lose some important information. So we consider assigning labels for each data.

#### 4.2.3. The Result of Label Assigned

This paper uses the probability estimate of classification algorithm to compute the probability of the data belonging to the class; if the probability is higher than the threshold we assign this class's label to the data. The process is shown in Algorithm 1.

**Figure 8:**
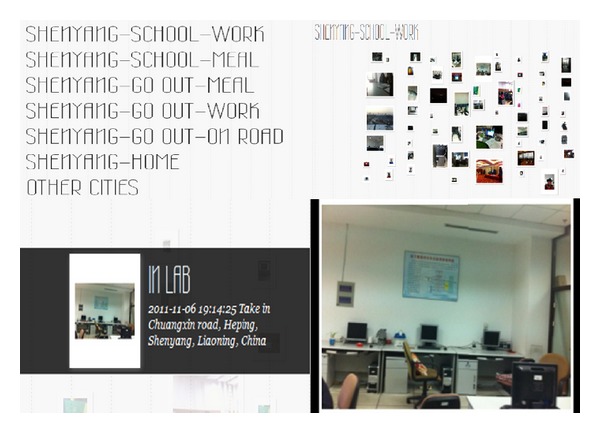
Application screenshots.

After we assigned labels to each data, this paper uses the labels to divide the data set and build the index. The structure is shown in Figure 6. In this way, this paper has sorted the data set and users can search and browse the data by the index. By analyzing this result we research the relationship between the user's important places and behaviors. The actual application scene of this method is shown in Figure 8.

## 5. Conclusions

In our study, we propose the method called geotagged photo data set based classification algorithm, which can build an index to quickly sort the data. This method has practical significance for the mobile terminal users to manage the photos. The result of classification can identify the user's important places and behaviors, so the study has a good research prospect.

## Figures and Tables

**Algorithm 1:**
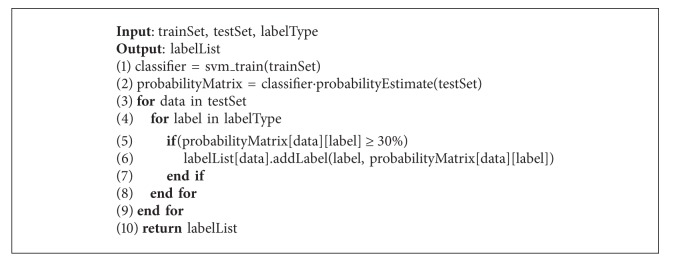
Label assigned.
